# Effectiveness of Eye Movement Desensitization and Reprocessing-EMDR Method in Patients with Chronic Subjective Tinnitus

**DOI:** 10.3390/brainsci14090918

**Published:** 2024-09-13

**Authors:** Fatih Bal, Muzaffer Kırış

**Affiliations:** 1 Department of Psychology, Sakarya University, Sakarya 54187, Türkiye; 2 Department of Ear, Nose and Throat Diseases, Ankara Yıldırım Beyazıt University, Ankara 06010, Türkiye; drkiris@hotmail.com

**Keywords:** tinnitus, EMDR, psychotherapy

## Abstract

This research aimed to investigate the effectiveness of the Eye Movement Desensitization and Reprocessing (EMDR) method on chronic subjective tinnitus. The research was planned as an observational study. The study group comprises individuals who applied to the training and research hospital in Ankara between 2019 and 2020 and were aged between 15 and 60 years old. They were identified as having tinnitus. The study samples were determined as 36 participants selected through purposeful sampling. The samples of the 36 participants included in the study. 12 were assigned to the 1st Group EMDR and Masking Group, 12 to the 2nd Group Masking and EMDR Group, and 12 to the 3rd Control Group. The study’s dependent variable was the tinnitus levels of the participants, and the independent variable was EMDR and the Masking method. The dependent variable data of the study was collected with the Visual Analog Scale and Tinnitus Handicap Inventory (THI). EMDR and Masking methods used as independent variables in the study were conducted in eight sessions for two months. As a result of the Wilcoxon Sign test used to determine whether the EMDR Method is effective on tinnitus severity level, the difference between tinnitus severity level pretest and post-test median scores of tinnitus patients was found to be statistically significant. Our research findings show that the EMDR method reduces and improves chronic subjective tinnitus, and further studies with a larger sample size could confirm our results.

## 1. Introduction

Tinnitus is the perception of sound without an external sound stimulus [1]. Its background can be divided into primary and secondary cases. Secondary cases include pathologies of the outer, middle, and inner ear. Tinnitus can be objective or subjective; the latter can only be identified by the sufferer. Previous research has shown that tinnitus significantly impacts quality of life and daily functioning [2]. Tinnitus negatively affects patients’ quality of life in many areas [3,4]. Many who complain of tinnitus patients primarily express their psychological concerns [3]. Tinnitus negatively affects people’s quality of life by disrupting the flow of their everyday life. Depression, hopelessness, anxiety, panic attacks, and behavior problems are the most common psychological problems in individuals with Tinnitus [5]. A study was conducted to evaluate the prevalence of auditory-vestibular symptoms among children following SARS-CoV-2 infection or vaccination for SARS-CoV-2. The findings indicated that children with a history of SARS-CoV-2 infection exhibited a higher prevalence of symptoms. However, no significant difference in symptom prevalence was observed in children with a prior diagnosis of unilateral hearing loss [2]. However, the correlation between tinnitus disability and psychiatric comorbidity indicates the importance of psychological factors in tinnitus management. Tinnitus disability has a stronger effect on depression scores. Tinnitus disability should be reduced to prevent the occurrence of comorbidity [6].

Tinnitus is caused by the abnormal perception of regular neural activity induced by pathology somewhere in the auditory system or even when there is no sound in the auditory system. Usually, such signals are suppressed by neural filtering in the brain stem. In tinnitus, the filtering system does not work correctly, and the functioning of the nerves in their everyday activity is perceived as sound at the brain level. Tinnitus perception is dependent on stress from attention and emotional state. Eye Movement Desensitization and Reprocessing (EMDR) activates neurobiological mechanisms by reprocessing information. This process provides physiological changes in the brain and facilitates information reprocessing [7,8]. EMDR reduces physical symptoms and chronic pain by combining unresolved trauma memories and somatic symptoms [9,10]. There are also similarities in the clinical treatment of phantom pain and phantom voice.

The absence of an effective method for the treatment of tinnitus presents the importance of the EMDR method in the treatment of tinnitus. With EMDR treatment, these physical memory and pain sensations can be treated. Tinnitus is known as phantom sound, and researchers have found an overlapping relationship between brain networks between tinnitus and phantom pain [3]. The brain networks play an essential role in revealing and preserving this phantom perception [11]. Besides being a significant health problem frequently seen in society, tinnitus causes various psychosocial issues in patients and significantly affects the individual’s quality of life. The cause of tinnitus is not fully known. Due to the uncertainties in the etiology, there are many treatment options whose efficacy has not yet been fully demonstrated. Many pharmacological agents have been tried in treating tinnitus, and research on many pharmacological agents is still being conducted. Surgical treatment is rarely used for tinnitus, and its place in tinnitus treatment is limited [12]. However, a standard treatment method that is thought effective for tinnitus has not been determined [13].

Pharmacological treatment has been demonstrated to be an effective intervention for acute cases of tinnitus, particularly when acute sensorineural hearing loss is present [14]. Furthermore, it would be beneficial to elucidate that the clinical manifestation of tinnitus is highly variable and that self-perceived emotional state also significantly influences treatment efficacy [15]. It thus appears that no pharmacological treatment is effective for all cases of tinnitus [16,17].

A history of trauma to the head, particularly to the ear and/or neck, is frequently associated with the onset of tinnitus. The most reported trigger for the development of chronic tinnitus in the general population is chronic or acute noise-related trauma [18]. In one study, ototoxicity resulting from immunosuppressive therapy was observed, with hearing loss representing the most prevalent clinical symptom. This was predominantly bilateral, while tinnitus was reported in over half of the participants. This indicates a potential correlation between tinnitus and hearing loss, ototoxicity, and immunosuppressive therapy [19]. Although tinnitus has traditionally been considered an otological disorder, recent advances in neuroimaging technologies and animal studies have proved it is linked to neuronal connections. The neuronal representation of tinnitus is constituted by an increased neuronal firing rate, enhanced neuronal synchronization, and changes in tonotopic organization in the central auditory pathways [20]. The pathophysiological mechanisms proposed to cause tinnitus encompass a range of hypotheses, from those pertaining to the cochlea, such as separation of stereocilia of hair cells and spontaneous otoacoustic emission, to those concerning the neural aspects, such as disruption of the spontaneous resting activity of primary auditory nerve fibers and Moller’s proposal of ephaptic conduction (“cross-talk”) between adjacent nerve fibers [21,22]. Many of these cases entail some form of disturbance to the peripheral auditory system, which can manifest as a range of hearing impairments. However, one of the most significant yet underappreciated causes of tinnitus is not related to the ear itself but to its connections with the auditory system within the central nervous system. Myofascial disorders of the head and neck have been demonstrated to induce tinnitus by regulating the activity of the somatosensory system, which projects to the central auditory system at multiple levels, including the dorsal cochlear nucleus (DCN) and inferior colliculus. Many of these cases entail some form of disturbance to the peripheral auditory system, which can manifest as a range of hearing impairments. However, one of the most significant yet underappreciated causes of tinnitus is not related to the ear itself but to its connections with the auditory system within the central nervous system. Myofascial disorders of the head and neck have been demonstrated to induce tinnitus by regulating the activity of the somatosensory system, which projects to the central auditory system at multiple levels, including DCN and the inferior colliculus [23].

The negative psychosocial effects of tinnitus on individuals are known [24]. Individuals who report tinnitus are more likely to experience depression, anxiety, and sleep disorders, and their quality of life decreases [25]. The mental health status of individuals with tinnitus is rated as worse than that of those without tinnitus [26]. Reports show that individuals with tinnitus and a psychological condition have a worse psychological state [27]. These conditions can interact with each other, exacerbating their effects. A bidirectional effect exists between response to tinnitus and an individual’s mental health status. The interaction between tinnitus and a psychological condition is particularly pronounced in individuals with psychological damage, such as post-traumatic stress disorder [28]. Trauma causes significant changes in the limbic system and may eventually cause tinnitus. Therefore, it is thought that tinnitus can be eliminated by reducing trauma in the limbic system through the EMDR method. According to studies on EMDR, EMDR is used as a helpful treatment method in treating symptoms that cannot be reduced despite regular treatments or in treating somatic diseases that cannot be treated medically. Studies show that EMDR can also reduce physical and psychological symptoms. EMDR is a treatment approach constantly evolving and integrating different contributions from major psychological orientations [29]. It aims to develop a robust and comprehensive treatment by incorporating aspects of various modalities, making it suitable for clinicians of all orientations. The adaptive information processing model, which proposes neurobiological underpinnings of experiential-based disorders, can potentially serve as an integrative theory for understanding and exploring clinical phenomena [30]. However, it should be noted that this model is not a method itself but rather a framework that can be used within different orientations. The hope is that further rigorous research will be conducted to validate the information processing formulation. Overall, EMDR provides a versatile approach that can be used within various therapeutic orientations [31,32]. In this study, it was investigated whether EMDR and Masking methods would be partially or completely effective in the treatment of chronic subjective tinnitus in individuals with tinnitus. This study aims to eliminate chronic subjective tinnitus by activating the disturbing information stored in the nervous system, which is thought to cause tinnitus due to past negative experiences, with the EMDR method.

The hypotheses of this study are organized as follows;

**H1:**  *The level of discomfort from chronic subjective tinnitus of individuals with chronic subjective tinnitus who are included in the EMDR method will decrease significantly compared to individuals who are not included in the practice.*

**H2:**  *EMDR method is positively more effective than Masking method on the level of discomfort from chronic subjective tinnitus in individuals who apply due to tinnitus.*

**H3:**  *There is a difference between the chronic subjective tinnitus levels in the groups in which EMDR and the Masking method are not applied to individuals who apply due to tinnitus.*

**H4:**  *The frequency and severity levels of tinnitus will decrease in the groups in which EMDR and the Masking method are applied in individuals who apply due to chronic subjective tinnitus.*

## 2. Material and Methods

Ethics committee permission was obtained (protocol code 2019/27). All participants signed informed consent. This research was conducted at the Ear Nose Throat (ENT) Clinic Hearing Speech Balance Diagnosis Treatment Center of the Training and Research Hospital in Ankara. In this research, 36 individuals aged 15–60 participated. A detailed medical history was obtained from the individuals who participated in the study; the content and purpose of the study were explained to all participants, and their written permissions were obtained.

### Participants and Procedure

The study group comprises individuals who applied to the training and research hospital in Ankara between 2019 and 2020 and were aged between 15 and 60 years old. In this study, subjects with subjective tinnitus complaints participated for at least three months. While selecting the study group for the research, one of the purposeful sampling methods was chosen according to the criterion sampling technique [33]. Criterion sampling studies all situations that meet a set of predetermined criteria. Participants were informed about the study’s aims and procedures and signed an informed consent form before participating.

The otoacoustic emissions and auditory brainstem responses of the participants were recorded at the ENT outpatient clinic. However, information about certain disorders, such as temporomandibular disorders, was not obtained.

The gender distribution in the study was as homogeneous as possible, with the inclusion of an equal number of male and female participants. Furthermore, cases presenting with unilateral or bilateral symptoms were also included. The location of the tinnitus symptom was determined through the administration of a questionnaire. In addition to indicating subjective tinnitus, participants were included in the study if their cases were acute or chronic. Furthermore, the study included individuals with secondary tinnitus or single tinnitus. For detailed information, please refer to Table 1. (The first group consisted of seven men and five women, while the second group consisted of nine men and three women. The third group consisted of six men and women in the control group. All of them were found to have chronic subjective tinnitus).

## 3. Ethics

Written informed consent was signed by all participants before data collection. The study was approved by the ethics committee of Ankara Yıldırım Beyazıt University with the number (201927) and was conducted in accordance with the Declaration of Helsinki.

### 3.1. Exclusion Criteria

ENT and audiological examinations were performed on all participants. Subjects who had a disease that could cause objective tinnitus, anatomical problems or acute disease related to the outer ear and middle ear, acoustic tumor, Ménière’s disease, ear surgery, neurotological intervention, neuropsychiatric problem, general physical fitness disorder, and social life were restricted for this reason, and whose written permission could not be obtained were not included in the study. The inclusion criteria were as follows: Subjective tinnitus diagnosis, age range between 18 and 60 years, normal ENT examination, no systemic health problems, normal hearing level, Type A tympanogram, and voluntary participation.

A pure-tone hearing test was conducted on all participants. Following the pure tone hearing test, the tinnitus frequency, intensity, minimal masking level (MMS), and residual inhibition were determined using pure tone, warble tone, pulsed noise, and broadband noise between 125 and 16,000 Hz.

In this study, data regarding the general characteristics of tinnitus were collected from the participants prior to the tinnitus mapping procedure. Information regarding the patient’s tinnitus was obtained through a series of questions, including the date the tinnitus began, the location of the sound, the source of the sound, whether the sound originated from the right or left ear, and a detailed description of the sound. Subsequently, all participants were informed individually. Tinnitus maps were established through clinical audiometry. The Tinnitus Map Interacoustics AC40 (Assens, Denmark) clinical audiometer and TDH-39 supra-aural headphones were employed for this purpose. The products used belong to Ankara Yıldırım Beyazıt Eğiitm and Research Hospital, a state hospital in Turkey.

Audiometric examinations were conducted in a standardized, quiet room utilizing an Interacoustic AC40 clinical audiometer and TDH-39 supra-aural headphones. The analysis encompassed both airborne hearing thresholds within the 125–8000 Hz range and bone conduction hearing thresholds within the 250–4000 Hz range, with all individuals participating in the study being included. The pure tone averages were calculated by taking the arithmetic mean of the hearing thresholds at 500, 1000, and 2000 Hz. The study included individuals presenting with tinnitus without hearing loss. The frequencies between 8000 and 16,000 Hz were analyzed using high-frequency headphones (KOSS HV/1A and Sennheiser HDA 200). Sennheiser is a company based in Germany and its products are recognized worldwide. The products used belong to Ankara Yıldırım Beyazıt Eğiitm and Research Hospital, a state hospital in Turkey.

Three groups were created for this study, carried out in two stages.

Group 1: Individuals who have chronic subjective tinnitus and received EMDR (EMDR and Masking Group) (12 participants),

Group 2: Individuals with chronic subjective tinnitus and Masking (Masking and EMDR Group) (12 participants)

Group 3: Individuals with chronic subjective tinnitus who did not receive therapy (Control Group) (12 participants).

In the first stage of the research, while EMDR and Masking were applied to the participants in the first Group, the Masking Group and EMDR were used on the participants in the second Group.

The study was conducted on a biweekly basis for a period of eight weeks, with each session lasting for 45 min. At the conclusion of the treatment period, patients were invited to participate in follow-up assessments and complete questionnaires. Additionally, scales were administered to each group. The participants completed the Tinnitus Handicap Inventory (THI) and Scale, as well as the Visual Analog Scale (VAS). The severity of tinnitus was evaluated using pre-test and post-test scores.

### 3.2. Measurements

#### 3.2.1. Tinnitus Handicap Inventory (THI)

Tinnitus Handicap Inventory (THI), a questionnaire developed by Newman et al. In 1996, consisting of 25 questions and scored between 0 and 100. THI is a questionnaire form that is a highly reliable test, and it is suitable for the age, gender, and hearing thresholds, is easy to apply, and provides more psychometrically specific measurements [13]. It has functional, emotional, and catastrophic sub-scores. The total score obtained from the items is 100. A study was conducted to assess the validity and reliability of the Turkish version of the test [34]. A correlation has been identified between the Tinnitus Disability Questionnaire and the Visual Analogue Scale, which is used for the assessment of tinnitus. The evaluation of the questionnaire is classified as follows: 0–16 points indicate very mild first degree, 18–36 mild second degree, 38–56 points moderate third degree, 58–76 points severe fourth degree, and 78–100 points very severe fifth degree [35].

#### 3.2.2. Visual Analog Scale (VAS)

The patient is asked to score his perception of tinnitus, discomfort from high volume, hearing loss, and quality of life in the range of 0–10. VAS is a test used frequently by different branches for all kinds of Pain and has various versions. It has been adapted and used for tinnitus in many studies. The visual analog scale shows the frequency, duration, and severity of tinnitus in patients and how uncomfortable patients are from tinnitus. In visible analog scale questions, the patient guides the level of subjectivity on a ruler numbered from 0 to 10, and 0 is shown in the happiest and 10 in the most unhappy manner. The visual analog scale is divided into five groups: tinnitus severity is shown with VAS-1, tinnitus duration, the frequency with VAS-2, disturbing level VAS-3, attention deficit with VAS-4, and sleep disorders with VAS-5 [36].

#### 3.2.3. Treatment Protocol

EMDR includes imaging the past experiences or traumatic experiences of the psychological counselor or therapist using finger gestures, providing the systematic movement of the client’s eyes in a systematic right-to-left direction. EMDR is a therapy approach that helps clients reprocess their negative life experiences to contribute to their emotional health [37].

Study participants received standard EMDR protocol. All eight stages described in this session are included [6]. Appropriate distance, position, safety, and silence room are provided for EMDR therapy. Each participant underwent a maximum number of 10 sessions of EMDR therapy lasting 45 min each. EMDR therapy sessions occurred regularly, with a frequency of once every 1 to 2 weeks. A single clinical psychologist administered the EMDR.

The study participant is then asked to create a description of their chronic subjective tinnitus that included: (1) an image or a felt sense that represents the study participant’s tinnitus experiences; (2) negative belief(s) in relation to the tinnitus experiences; (3) a preferred belief in relation to the experiences; (4) the (usually negative/undesirable) emotions associated with the experiences; and (5) the physical sensations associated with the experiences. The therapist first elicits a disturbing memory from the participant, and the participant is asked to rank the disturbance of that memory on a scale ranging from 0 to 10 (0 = no disturbance; 10 = worst disturbance imaginable). At various stages in the processing, the therapist checks in with the client to gauge the client’s level of disturbance. The goal of EMDR processing is to bring the Subjective Units of Disturbance (SUD) down to 0 or a one and to keep it maintained at that level. Participants were asked to form a negative opinion about the tinnitus severity of their tinnitus if SUD is not taken. Subsequently, patients were asked to produce alternative positive thoughts and demonstrate how much they believed on the Validity of positive Scale (VOC) (1 is completely false and seven is completely true). Afterward, patients were asked to visualize images depicting trauma focusing on negative thoughts and experiencing emotions and physical sensations.

An EMDR therapy session is an individual therapy session with a trained EMDR therapist. Prior to the initial EMDR session, each study participant was provided with a verbal and written explanation of the rationale behind the use of EMDR therapy for their tinnitus. EMDR was provided according to the standard eight-phase protocol comprising (1) history and treatment planning, (2) client preparation, (3) assessment, (4) desensitization, (5) installation, (6) body scan, (7) closure, and (8) reevaluation. Each study participant worked with the therapist to collect relevant history and current information about the study participant’s experiences of their tinnitus, which provided the basis for an individually tailored formulation.

##### Tinnitus Masking Therapy (TMT)

All participants included in the study were administered THI at the beginning of the study. As a result of THI, tinnitus frequency, intensity, MMS measurement, and residual inhibition were performed as tinnitus assessment tests. For tinnitus frequency matching, two different pure tones, one low frequency and one high frequency, were sent to the contralateral ear at various frequencies at a level audible to the participant. The patient was asked to select the one closest to the tinnitus frequency from these two pure tones. If the patient resembled the low-frequency sound, the tinnitus frequency was determined by sending two different pure tones again, one low-frequency sound in addition to the frequency they had previously resembled, and one high-frequency sound in addition to the frequency they had previously resembled, if the patient resembled the high-frequency sound. This process was continued until the tinnitus frequency was determined. After the tinnitus frequency was determined, in unilateral tinnitus, two pure tones with two different intensities were sent to the contralateral ear at the previously determined tinnitus frequency, 10 dB lower and 10 dB higher than the intensity level that the participant could hear comfortably. The participant was asked which sound was closer to their tinnitus, and the procedure was continued until the tinnitus intensity level was found. The minimum masking level was determined by sending a pulsed noise to the participant’s contralateral ear at an intensity level 5 dB lower than the tinnitus intensity level. The pulsed noise was increased by 5 dB until the level at which the participant could not detect tinnitus was determined. The minimum intensity level of the pulsed noise required to prevent the participant from hearing their tinnitus was defined as the minimum masking level. To investigate residual inhibition, the narrowband noise was presented for 60 s at the tinnitus frequency, 10 dB above the minimal masking level. The participant was asked whether there was a decrease or disappearance of tinnitus. Complete residual inhibition was defined as the disappearance of tinnitus, while partial residual inhibition was defined as the temporary disappearance of tinnitus after masking. After these procedures, the Masking method was applied.

### 3.3. Analysis of Data

The total number of the study group participating in the research is 36. Each group was assigned 12 individuals with tinnitus. Nonparametric statistical methods are used when the number of participants in the study is below 30 [38]. Therefore, nonparametric tests were used to analyze the data of the study. In addition, normality analyses were performed for each scale. In this study, Mann–Whitney U Test was used to determine the difference between the pre-test and post-test of the groups, and Wilcoxon Signed Rank Order Test was used to evaluate the pre-test and post-test differences within the groups. The data collected in the study were analyzed on the computer using SPSS 25 (Statistical Programme for Social Sciences).

## 4. Results

Thirty-six patients with chronic subjective tinnitus were evaluated at the end of the measurements. Of those, twelve participants in 1st Group EMDR and Masking and Making and EMDR, 2nd Group Masking and EMDR, and 3ng Control were presented below.

Table 1 indicated the sex of the participants in 1. Group EMDR and Masking in the study: 58% are male and 42% are female. The marital status of the participants is 83% married, and 17% are single. It is seen that 25% of the child variable owned has no children, 8% have one child, 42% have two children, and 25% have three children. When the profession variable can be examined, 8% are retired, 25% are officers, 17% are students, 8% are in other professions, 33% are housewives, and 8% are self-employed. The age of the participants is 17% between the ages of 15–20, 8% between the ages of 21–30, 17% between the ages of 31–40, 25% between the ages of 41–50, and 33% between the ages of 51. and above. 2. Group Masking and EMDR participants are presented in Table 1.

When gender, number of children, and the variable are considered, it is seen that the groups are homogeneous in the 1st group EMDR and Masking and the 2nd group masking and EMDR groups within and between the groups (*p* > 0.001). On the other hand, in the marital status and occupation variables, it was determined that the groups were homogeneous in the 1st group EMDR and Masking (*p* > 0.001), and the groups were not homogeneous in the 2nd group masking and EMDR (*p* < 0.001).

Table 2 indicated that it was seen that 58% of chronic subjective tinnitus was caused by family problems, 33% from trauma, and 8% from other causes. The similarity of the sound of chronic subjective tinnitus was stated that 10% was wind, 50% humming, 10% motor, 20% water, and 10% ultrasound. When looking at the beginning of chronic subjective tinnitus, 25% of patients stated that chronic subjective tinnitus existed for one year, 8% for two years, 17% for three years, and 50% for five years or more. When the localization of chronic subjective tinnitus is examined, it is stated that 25% of them are chronic subjective tinnitus in the right ear, and 75% of them are bilateral. He stated that 100% of the patients had no hearing loss. Socio-demographic results of 2nd Group Masking and EMDR were presented in Table 2.

Looking at the cause, onset, similarity, and location of the tinnitus, it can be seen that the groups are homogeneous within and between the groups in the 1st group EMDR and masking and in the 2nd group masking and EMDR (*p* > 0.001). On the other hand, it can be seen that family problems are more common in the 1st group EMDR and masking in terms of causes of tinnitus, buzzing is the highest in terms of similarity, onset is more than five years, and location is in both ears.

According to Table 3, the total THI score of patients undergoing EMDR was 54.33 ± 19.39, and the total THI score of patients undergoing masking was 24.42 ± 19.69. The total VAS score of the patients who underwent EMDR was 25.50 ± 4.08, and the total VAS score of the patients who underwent masking was 8.83 ± 6.31. The total THI score of the patients in the 2nd group who underwent masking was 51.00 ± 24.23, and the total THI score of the patients who underwent EMDR was 51.67 ± 23.57. The total VAS score of the patients who underwent masking was 25.58 ± 2.78 and the total THI score of the patients who underwent EMDR was 24.33 ± 5.48. The total THI score of the patients in the 3rd group who were not masking with EMDR was 48.83 ± 24.80, and the total VAS score was 18.92 ± 6.13.

**Figure 1:**
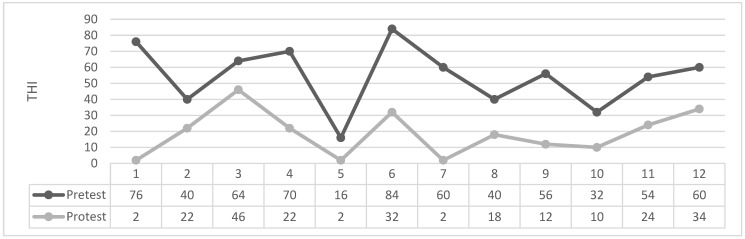
1. Group EMDR.

**Figure 2:**
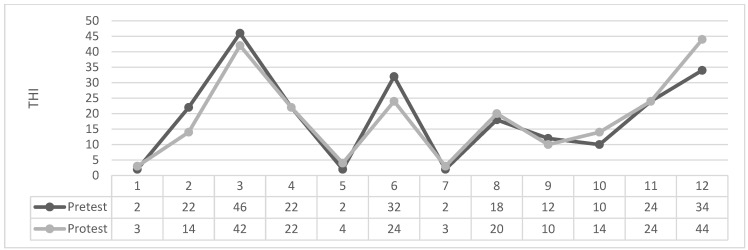
1. Group masking.

As can be seen in Table 4 and Figure 1, Figure 2, Figure 3 and Figure 4 the Wilcoxon Sign Test was applied to determine whether EMDR method was effective on tinnitus severity level in patients with tinnitus in Groups 1 and 2.

Figures: Participants’ individual THI Score Change Graph.

The Wilcoxon signed-rank test showed that in 1. Group EMDR produced a statistically significant change in THI total scores (Z = −3.062 *p* < 0.002). Indeed, the median THI score assessment was 54.33 (IQR 55) before treatment and 24.83 (IQR 22) after treatment.

The Wilcoxon signed-rank test showed that in 1. Group Masking produced a statistically no significant change in THI total scores (Z = −0.052 *p* > 0.959). The median THI score assessment was 24.41 (IQR 21) before treatment and 24.33 (IQR 21) after treatment.

The Wilcoxon signed-rank test showed that in 2. Group Masking produced a statistically no significant change in THI total scores (Z = −1.284 *p* > 0.189). The median THI score assessment was 51.00 (IQR 53) before treatment and 53.66 (IQR 60) after treatment.

The Wilcoxon signed-rank test showed that in 2. Group EMDR produced a statistically significant change in THI total scores (Z = −3.061 *p* < 0.002). The median THI score assessment was 51.66 (IQR 58) before treatment and 14.16 (IQR 22) after treatment.

The Wilcoxon signed-rank test showed that in 3. Group Control produced a statistically no significant change in THI total scores (Z = −423 *p* > 0.673). The median THI score assessment was 48.83 (IQR 49) before treatment and 50.25 (IQR 47) after treatment.

The Wilcoxon signed-rank test showed that 1. Group EMDR produced a statistically significant change in VAS total scores (Z = −3.063 *p* < 0.002). Indeed, the median THI score assessment was 25.50 (IQR 26) before treatment and 7.83 (IQR 6) after treatment.

The Wilcoxon signed-rank test showed that in 1. Group Masking produced a statistically no significant change in VAS total scores (Z = −1.632 *p* > 0.103). The median THI score assessment was 8.33 (IQR 7) before treatment and 8.33 (IQR 21) after treatment.

The Wilcoxon signed-rank test showed that in 2. Group Masking produced a statistically no significant change in THI total scores (Z = −1.342 *p* > 0.180). The median THI score assessment was 25.58 (IQR 27) before treatment and 53.66 (IQR 26) after treatment.

The Wilcoxon signed-rank test showed that in 2. Group EMDR produced a statistically significant change in THI total scores (Z = −3.063 *p* < 0.002). The median THI score assessment was 24.33 (IQR 25) before treatment and 8.50 (IQR 9) after treatment.

The Wilcoxon signed-rank test showed that in 3. Group Control produced a statistically no significant change in THI total scores (Z = −423 *p* < 0.673). The median THI score assessment was 18.91 (IQI 21) before treatment and 18.58 (IQI 21) after treatment.

These results indicated that the EMDR method has an important effect in decreasing THI total scores. On the other hand, the Masking method did not have a significant effect on reducing THI total scores. Similar results were seen in the VAS total scores.

According to Table 5, the Mann–Whitney U test was used to determine the difference between the total THI scores of the 1st group treated with EMDR and Masking method and because of the test, the difference between the THI scores of the patients treated with EMDR and Masking method was found. The difference was statistically significant (U = 11,000, *p* < 0.005). Looking at the rank means, it is understood that the total THI scores of the participants treated with the EMDR method (21.11, IQR 3) were lower than the THI rank mean of the participants treated with the masking method (37.00, IQR 3). This finding shows that the EMDR method is more effective than the masking method in reducing the participants’ THI scores.

According to Table 5, Mann–Whitney U test was used to determine the difference between the total THI scores of the 2nd group masking treated with EMDR and Masking method and as a result of the test, the difference between the THI scores of the participants treated with EMDR and Masking method was found. The difference was statistically significant (U = 14,000, *p* < 0.005). Looking at the rank means, it is understood that the total THI scores of the participants treated with the EMDR method (13.89, IQR 7) were lower than the THI rank mean of the participants treated with the masking method (33.29, IQR 3). This finding shows that the EMDR method is more effective than the masking method in reducing the participants’ THI scores.

According to Table 5, Mann–Whitney U test was used to determine the difference between the total THI scores of the 1st group masking treated with EMDR and 3. Group Masking method, and as a result of the test, the difference between the THI scores of the participants treated with EMDR and Masking method was found. The difference was statistically significant (U = 16,000, *p* < 0.005). Looking at the rank means, it is understood that the total THI scores of the participants treated with the EMDR method (16.77, IQR 7) were lower than the THI rank mean of the participants treated with the masking method (38.29, IQR 28). This finding shows that the EMDR method is more effective than the masking method in reducing the participants’ THI scores. Other comparisons and VAS results are shown in Table 5.

## 5. Discussion

This study aims to examine the effectiveness of the EMDR method on tinnitus. Our research found that the level of tinnitus in individuals with tinnitus showed a significant improvement after the EMDR method. In contrast, the Masking method did not significantly improve tinnitus. It was concluded that applying the EMDR method would be effective in individuals with subjective tinnitus. The EMDR method, the subject of our research, is an advanced version of the CBT method and includes the CBT protocols. This integrative psychotherapy method includes bilateral stimulation, such as rapid eye movement from side to side. As a psychotherapy method, EMDR is gaining popularity as an increasingly effective treatment option. Philips et al. [39] similarly found that EMDR therapy effectively reduced tinnitus. In our study, the participants’ symptoms were reduced after EMDR application, but this reduction was observed at different levels among the subjects. In the control group, no statistically significant change in VAS and THI scores was applied simultaneously with the experimental group. These results suggest that the therapy used in individuals with tinnitus is beneficial. It can be concluded that EMDR therapy has a significant effect on treating tinnitus in individuals with it.

The initial hypothesis of this research project is that the tinnitus scores of individuals with tinnitus who undergo EMDR therapy will demonstrate a notable reduction when compared to the tinnitus scores of individuals who do not participate in the therapy. In formulating this hypothesis, we used the tenets of Shapiro’s Adaptive Information Processing (AIP) model. Given that traumatic experiences impede the normal functioning of the information processing system in our nervous system, the information is stored in a manner that reflects its initial experience. Even after a few years, the data remain stored without processing. Consequently, the information in question becomes frozen over time. Therefore, it persists within the neural network. This study aims to assess the efficacy of EMDR as a treatment for chronic subjective tinnitus. Two experimental studies have demonstrated that EMDR is an efficacious treatment for tinnitus distress, with the effect maintained at the three-month follow-up [40]. The study indicates that EMDR is an efficacious treatment for tinnitus, with bimodal treatment EMDR resulting in a reduction in tinnitus complaints [41]. Furthermore, EMDR has effectively treated comorbid conditions, such as tinnitus and hyperacusis [39]. A notable decrease in THI scores was observed following EMDR treatment, with this improvement sustained over an extended period. The working mechanisms are thought to include a working memory account, classical conditioning, physiological changes associated with the orienting response, and neural integration theories. However, the most explanatory model remains to be determined. The findings of this study indicate that different therapeutic interventions integrate over time and reflect progress in the treatment process. However, no significant between-group differences were observed on specific scales after treatment [39]. These findings are consistent with those of our own study.

At present, no specific pharmaceutical agent has been approved for the treatment of tinnitus. Several potential therapeutic modalities have been shown to provide patients with varying degrees of symptomatic improvement and an enhanced quality of life. In the early stages, sedatives and other medications may be beneficial. Nevertheless, research into more effective treatments is ongoing. A variety of methods have been demonstrated to be effective in the treatment of tinnitus. These include prophylaxis, medical therapy, surgical therapy, electrical stimulation, the use of hearing aids, the application of tinnitus maskers, tinnitus reeducation therapy, psychological support, hypnosis, and the use of muscle relaxants and relaxation practices. The treatment above protocols, which include attention control, biofeedback therapy, cognitive behavioral therapy (CBT), auditory discrimination training, laser applications, acupuncture therapy, and ozone therapy, are the most employed treatment options for tinnitus. The adverse effects of tinnitus on quality of life can be mitigated through a trained professional’s administration of CBT sessions.

Following the Jastreboff theory, the initial stage in the development of tinnitus is the supervisory region, which is then followed by the limbic system. The limbic system is responsible for the emotional response to tinnitus and the operation of the autonomic nervous system. The dysfunction of the autonomous and limbic systems can account for the prevalence of anxiety, depression, and sleep disorders among individuals with tinnitus. It has been demonstrated that exposure to physical and emotional stress in the perception of tinnitus can potentially disrupt the tinnitus suppression mechanisms by influencing the limbic and autonomic systems. Therefore, tinnitus is sustained by the autonomic system and activates the autonomic system [36]. Traumatic personal experiences can cause chronic tinnitus [37]. This study was designed with the hypothesis that the EMDR method can treat tinnitus by eliminating trauma in the limbic system by activating the suppression of tinnitus through the suppression of the limbic system and the suppression mechanisms of tinnitus. In conclusion, the findings of our research corroborate this hypothesis. Our study hypothesized that the EMDR method would eliminate trauma in the limbic system and activate the tinnitus suppression mechanisms, thereby eliminating tinnitus. This was based on the supposition that the limbic system is suppressed due to trauma and that tinnitus suppression mechanisms are canceled. Indeed, the findings of our study substantiate this hypothesis. In a study by Philips et al. [39], individuals diagnosed with tinnitus were administered a 10-session EMDR treatment. The study demonstrated that EMDR resulted in most participants’ clinically and statistically significant improvement in tinnitus symptoms. Furthermore, the beneficial effects of the treatment were observed to persist for six months following the conclusion of the intervention. These findings are consistent with those of our study.

In a separate study, the efficacy of EMDR and CBT was evaluated in the context of subjective tinnitus, specifically in relation to tinnitus phantom perception. The findings of this study indicate that EMDR may be a valuable approach for treating individuals with subjective and chronic tinnitus. These results align with those of our research. The EMDR protocol entailed eight stages and was conducted over six weeks, with each session lasting 90 min. Eye movements were employed during EMDR sessions, and additional tactile stimulation was utilized in seven cases. In the initial three to five sessions, memories associated with the tinnitus were processed. In the concluding sessions, the subject was instructed to engage with the tinnitus sensations evoked by the EMDR technique. In this study, all sessions were recorded on video to provide feedback. Following an examination of 43 of the 226 patients seen by otorhinolaryngologists, treatment was initiated with 35 participants. A total of 35 participants were included in the study. Five participants discontinued the treatment regimen at varying stages and for disparate reasons. Three participants exhibited no subjective tinnitus complaints following three to five EMDR sessions. These findings are consistent with those of our previous study.

The second hypothesis of our research is that the EMDR method is more effective in reducing tinnitus levels in participants than the masking method in individuals who apply for tinnitus. The primary objective of formulating this hypothesis is to demonstrate the efficacy of EMDR in addressing tinnitus and examine its impact on tinnitus. The concept of masking as a method for suppressing tinnitus was first proposed by Jones and Knudsen in 1928 [42]. Subsequently, Saltzman and Ersner advanced the use of hearing aids for masking [43]. By the late 1970s, masking had emerged as a successful approach to treating symptomatic tinnitus [44]. The objective of masking is to displace the tinnitus by providing an external sound to the participants, thereby eliminating the perception of tinnitus. To identify the most efficacious spectrum for masking, considerable attention has been devoted to the psychoacoustic characteristics of the masking sound, including its frequency and intensity. Diagnostic studies have concentrated on tinnitus intensity, frequency, and maskability. However, this did not affect the ability to predict tinnitus intensity, frequency, and maskability results. Furthermore, it was not deemed sufficient to elucidate why tinnitus of an identical nature induces discomfort at disparate levels in disparate individuals [45].

Consequently, the tinnitus severity level of the participants who received the initial group masking method was determined to be higher than the tinnitus level of the participants who received the EMDR method. In other words, the tinnitus level of participants who received the EMDR method was lower than that of participants who received the masking method. The results demonstrate that the EMDR method is more efficacious in treating tinnitus than the masking method. In a study by Marcus, Marquis, and Sakai, the EMDR method is more effective in recovery than other therapy methods [46]. As evidenced by our findings, the EMDR method is more effective on tinnitus than the Masking method.

Our study’s findings indicate no statistically significant difference between the pretest and post-test scores of the Masking method in participants with tinnitus in the first and second groups. In other words, applying the Masking method to Groups 1 and 2 did not result in any discernible improvement or reduction in the participants’ tinnitus. It was therefore observed that the application of the Masking method in the first and second group participants did not change the tinnitus level of the participants.

The Masking method presents several challenges. The masking method is not straightforward for some participants. It was found that approximately 50% of tinnitus participants were unable to mask their tinnitus. Additionally, participants with hypersensitivity to sound cannot use the Masking method, as the device’s loudness exceeds their discomfort levels and may exacerbate their symptoms. Following the masking method’s application, most participants encountered difficulties in re-establishing their pre-existing perception of tinnitus. Accordingly, the external sound must be amplified continuously to suppress the tinnitus sound. This may result in an exacerbation of the tinnitus sound. Consequently, the masking method employed to mitigate tinnitus may exacerbate the perception of the tinnitus sound due to the treatment administered to the participants. An improvement of up to 58% was observed only after an effective masking process. It is essential to exercise caution when selecting participants, as prolonged use may potentially result in cochlear damage. It was emphasized that the etiology of Hazell is an important consideration in tinnitus masks [47]. While the treatment is highly effective in participants with Ménière’s disease, it is less beneficial in those with noise-related hearing loss. It was stated that the Masking method treatment should not be expected to yield immediate benefits and that participants will demonstrate progress after applying the treatment for at least two or three months.

In some participants, the persistence of tinnitus lends support to the concept of ‘central tinnitus’ despite the damage to the inner ear and the severing of the eighth cranial nerve. Central tinnitus is typically not localized, whereas peripheral tinnitus may be localized in both ears. The primary identified causes of central tinnitus are lesions, inflammations, and vascular anomalies. Consequently, the Masking method is frequently ineffective in suppressing tinnitus. The third hypothesis of our research is that there is no significant difference between tinnitus levels in the groups that did not apply the EMDR method and the masking method in individuals who used it for tinnitus. This hypothesis was formulated to detect changes in tinnitus levels in groups that did not apply the EMDR and masking methods. The results of the study demonstrated that there was no statistically significant difference between the pretest and post-test mean scores of the participants in the third group with tinnitus who did not apply EMDR and masking. In other words, it was determined that there was no regression or reduction in tinnitus in participants who received the EMDR and masking method, which was not applied to the third group. Consequently, no alteration was discerned in the tinnitus levels of the participants in the third group who did not utilize the EMDR and masking techniques.

The fourth hypothesis of our research is that there will be a reduction in tinnitus frequency and severity levels in groups that apply EMDR and masking methods to individuals who apply for tinnitus. The results of our study demonstrated a statistically significant difference between the frequency and severity levels of the participants in the first and second groups who underwent EMDR treatment and the mean scores of the pretest and post-test scores. A statistically significant difference was identified between the frequency and severity levels of the first and second-group participants who underwent masking therapy. No statistically significant difference was identified between the frequency and severity levels of the third group participants, who did not receive either the EMDR or masking methods, in their pretest and post-test scores. The results demonstrated that the EMDR method reduced tinnitus frequency and severity levels among participants. Conversely, the masking method did not decrease tinnitus frequency and severity levels among participants. Furthermore, no change in participants’ frequency and severity levels were not subjected to the EMDR and masking methods. A literature review reveals that the limbic and autonomic systems have a beneficial effect on tinnitus, reducing its frequency and severity [48]. Given that the EMDR method employed in this study improves tinnitus by influencing the limbic and autonomic systems, it is aligned with the findings of studies investigating these systems’ impact on tinnitus.

The following results were obtained from the study conducted to examine the efficacy of the EMDR method on the tinnitus of 36 participants. Descriptive statistics: the tinnitus level post-test results obtained from the THI and VAS of the participants who underwent EMDR in the first group demonstrated a reduction in severity compared to the pre-test results. No difference was observed between the pre-and post-test tinnitus level results obtained from the THI and VAS results of the participants in the first group who underwent masking. The tinnitus level post-test results obtained from the THI and VAS results of the participants who underwent EMDR in the second group were lower than the pre-test results. No difference was observed between the tinnitus level pre-test and post-test results obtained from the THI and VAS results of the participants in the second group who underwent masking. The THI and VAS results for the third group of participants who did not undergo EMDR or masking. No significant difference was observed between the pre-test and post-test results for tinnitus level. Regarding the frequency of the tinnitus, the application of EMDR proved to be an effective approach for the participants in the first and second groups. The application of masking did not affect the tinnitus frequency level of participants in the first and second groups. The post-test tinnitus frequency level of the participants who applied the masking method in the experimental group was higher than that of the participants who underwent EMDR application. The tinnitus frequency level of the participants who did not receive any therapy was higher than that observed in the first group, who received both EMDR and masking. The tinnitus frequency level of the participants who did not receive therapy was higher than that of the second group, who received EMDR.

Regarding tinnitus severity, the application of EMDR is a viable option for participants in both the first and second groups. The application of masking did not result in a change to the tinnitus severity level of participants in the first and second groups. The post-test tinnitus severity level of the participants who applied the masking method was higher than those who underwent the EMDR application. The tinnitus severity level of the participants who did not receive any form of therapy was found to be higher than that of the participants in the first group who received either EMDR or masking. The tinnitus severity level of the participants who did not receive therapy was higher than that of Group 2, who received EMDR. Regarding the visual analog scale (VAS), it can be stated that EMDR is an effective method for treating tinnitus. The application of masking did not affect the visual analog level in Groups 1 and 2. The visual analog level of the participants who applied the masking method in the group was higher than that of those who underwent EMDR application. The visual analog level of the participants who did not receive therapy was higher than that of the participants in Group 1 who received EMDR and masking. The visual analog level of the participants who did not receive therapy was higher than that of the participants in Group 2 who received EMDR.

As is the case with all research, this study has limitations. The study’s findings indicated that the prevalence of tinnitus exhibited by participants varied in accordance with the number of sessions. While some participants exhibited a response in the initial session, others demonstrated a change in their condition after four or more sessions. The study was conducted over two months. It is therefore recommended that the duration of the sessions be organized as 10 or more sessions to facilitate the monitoring of changes resulting from the EMDR method in chronic subjective tinnitus. Moreover, the number of participants in the study was relatively small. It is therefore recommended that further research be conducted with larger study groups. The number of samples is a limitation of the study. It is therefore recommended that future multicenter studies employ larger sample sizes and longer treatment durations to gain a more robust understanding of the efficacy of the EMDR method in treating chronic subjective tinnitus.

The impact of EMDR on chronic subjective tinnitus was observed in each subject within the study. However, it should be noted that the change in chronic subjective tinnitus levels differed between individuals. The study was conducted with a total of 36 participants. Regarding the generalizability of the present study, it is recommended that it be conducted in a different environment with a larger sample size. Therefore, the study’s small sample size, lack of blinding, and lack of objective outcome criteria are limitations. In the present study, EMDR was applied exclusively to individuals presenting with subjective chronic tinnitus. For this reason, it may also be attempted in individuals with objective tinnitus.

## 6. Conclusions

Our research findings show that the EMDR method reduces and improves chronic subjective tinnitus. EMDR method is recommended as a treatment method for chronic subjective tinnitus and as an alternative treatment.

## Figures and Tables

**Figure 3:**
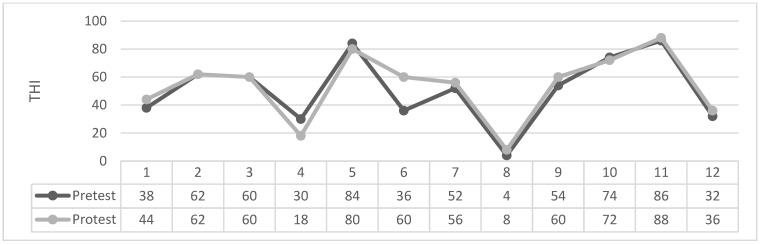
2. Group masking.

**Figure 4:**
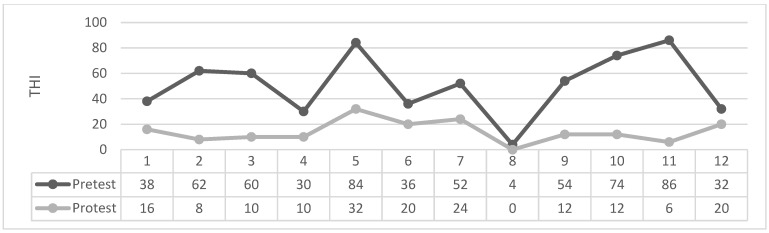
2. Group EMDR.

**Table 1:** Socio-demographic descriptive analysis results of the participants.

Variables		1. Group EMDR and Masking				2. Group Masking and EMDR			
		n	%	X^2^	*p*	n	%.	X^2^	*p*
Sex	Male	7	58	0.333	0.564		75	3.000	0.083
	Female	5	42			3	25		
	Total	12	100			12	100		
Marital status	Married	10	83	5.333	0.021	10	83	5.333	0.021
	Single	2	17			2	17		
	Total	12	100			12	100		
Number of children	None	3	25	2.667	0.446	2	17	2.000	0.572
	1 Child	1	8			3	25		
	2 Children	5	42			2	17		
	3 Children	3	25			5	42		
	Total	12	100			12	100		
Job	Retired	1	8	4.000	0.549	x *	X *	11.333	0.023
	Officer	3	25			2	17		
	Student	2	17			1	8		
	Other	1	8			1	8		
	Housewife	4	33			1	8		
	Self-Employment	1	8			8	58		
	Total	12	100			12	100		
Age	15–20 age	2	17	2.167	0.705	x	x	4.457	0.198
	21–30 age	1	8			1	8		
	31–40 age	2	17			3	25		
	41–50 age	3	25			6	50		
	51 age and up	4	33			2	17		
	Total	12	100			12	100		

* no retired participants in 2. Group Masking and EMDR.

**Table 2:** Socio-demographic descriptive analysis results of the participants.

		1. Group EMDR and Masking				2. Group Masking and EMDR			
Variables		n	%	X^2^	*p*	n	%	X^2^	*p*
Causality	Family problems	7	58	4.500	0.105	3	25	3.000	0.83
	Trauma	4	33			x *	X *		
	Other	1	8			9	75		
	Total	12	100			12	100		
Similarity	Wind	1	10	6.000	0.199	x	x	8.727	0.190
	Buzzing	5	50			5	45		
	Engine	1	10			1	9		
	Water	2	20			1	9		
	Ultrason	1	10			x	x		
	Bird	x	x			1	9		
	Bell	x	X			1	9		
	Whistle	x	x			1	9		
	Insect	x	X			1	9		
	Total	10	100			11	100		
Onset	One year	3	25	4.500	0.105	6	50	3.000	0.083
	Two years	1	8			3	25		
	Three years	2	17			1	8		
	Four years	x	x			1	8		
	Five years and up	6	50			1	8		
	Total	12	100			12	100		
Localization	Right	3	25	3.000	0.083	7	58	3.500	0.174
	Bilateral	9	75			3	25		
	Left	x	x			2	17		
	Total	12	100			12	100		
Hearing loss	−	0	0	8.333	0.004	0	0	8.333	0.004
	+	12	100			12	100		
	Total	12	100			12	100		

* represents no trauma participant in 2. Group Masking and EMDR, + represents hearing loss, − Represents no hearing loss.

**Table 3:** Tinnitus Handicap Inventory (THI) and Visual Analog Scale (VAS) descriptive analysis results of groups.

	Groups									
	Group 1 EMDR		Group 1 Masking		2. Group Masking		Group 2 EMDR		3. Group Therapy Not Applied	
	Mean	SD	Mean	SD	Mean	SD	Mean	SD	Mean	SD
THI Functional	21.33	9.04	11.50	8.74	21.33	0.73	21.67	12.24	21.83	12.49
THI Emotional	21.33	7.92	8.92	8.08	17.83	9.51	18.83	8.46	18.33	9.06
THI Catastrotrophic	11.67	6.14	4.00	3.81	11.83	4.93	11.17	4.93	8.67	5.28
Total THI score	54.33	19.39	24.42	19.69	51.00	24.2	51.67	23.57	48.83	24.80
Total VAS Score	25.50	4.08	8.33	6.31	25.58	2.78	24.33	5.48	18.92	6.13

**Table 4:** The results pre-test pro-test of Wilcoxon signed rank test of Tinnitus Handicap Inventory (THI) and Visual Analog Scale (VAS).

Scales	Groups Pre-Pro Test		Median	IQR	Decrease from THI1 to THI2	z	*p*-Value
THI	1. Group EMDR	THI Pre-test	54.33	55	55%	−3.062	0.002
		THI Pro-test	24.83	22	22%		
	1. Group Masking	THI Pre-test	24.41	21	21%	−0.052	0.959
		THI Pro-test	24.33	21	21%		
	2. Group Masking	THI Pre-test	51.00	53	53%	−1.284	0.199
		THI Pro-test	53.66	60	60%		
	2. Group EMDR	THI Pre-test	51.66	58	58%	−3.061	0.002
		THI Pro-test	14.16	12	12%		
	3. Group Control	THI Pre-test	48.83	49	49%	−0.423	0.673
		THI Protest	50.25	47	47%		
VAS	1. Group EMDR	VAS Pre-test	25.50	26	26%	−3.063	0.002
		VAS Protest	7.83	6	6%		
	1. Group Masking	VAS Pre-test	8.33	7	7%	−1.632	0.103
		VAs Pro-test	8.33	7	7%		
	2. Group Masking	VAS Pre-test	25.58	27	27%	−1.342	0.180
		VAS Pro-test	24.50	26	26%		
	2. Group EMDR	VAS pre-test	24.33	25	25%	−3.063	0.002
		VAS Pro-test	8.50	9	9%		
	3. Group Control	VAS Pre-test	18.91	21	21%	−1.632	0.102
		VAS Pre-test	18.58	21	21%		

Wilcoxon Signed Ranks Test/Based on positive ranks; THI: Tinnitus Handicap Inventory /VAS: Visual Analog Scale. n = 12.

**Table 5:** Mann-Whitney U results of Tinnitus Handicap Inventory (THI) scores of groups with and without EMDR method and masking method.

Scales	Groups	n	Median	IQR	Min.	Max.	Mean Rank	Sum of Rank	U	*p*-Value
THI	1. Group EMDR	12	21.11	3	1	6	7.42	89.50	11,000	0.002
	1. Group Masking	12	37.00	24	1	6	17.58	211.50		
	Total	24								
	2. Group Masking	12	33.29	26	1	6	16.50	198.00	14,000	0.023
	2. Group EMDR	12	13.89	7	1	5	8.50	67.00		
	Total	24								
	1. Group EMDR	12	16.77	6	1	6	21.22	94.00	16,000	0.007
	3. Group masking Control	12	38.77	28	1	6	45.50	176.00		
	Total	24								
VAS	1. Group Masking	12	32.44	24	1	5	32.33	178.00	20,000	0.504
	1. Group EMDR	12	35.45	23	1	6	32.67	162.00		
	Total	24								
	2. Group Masking	12	34.71		1	6	33.17	128.00	24,000	0.607
	3. Group EMDR	12	41.22		1	6	31.83	132.00		
	Total	24								
	2. Group Masking	12	23.331		1	6	18.83	126.00	21,000	0.012
	3. Group Control	12	41.229		1	6	36.17	134.00		
	Total	24								

## Data Availability

The data that support the findings of this study are available from the corresponding author upon reasonable request. The data are not publicly available due to privacy and ethical restrictionas.
